# Adolescents with Obstructive Sleep Apnea Adhere Poorly to Positive Airway Pressure (PAP), but PAP Users Show Improved Attention and School Performance

**DOI:** 10.1371/journal.pone.0016924

**Published:** 2011-03-17

**Authors:** Dean W. Beebe, Kelly C. Byars

**Affiliations:** 1 Cincinnati Children’s Hospital Medical Center, Cincinnati, Ohio, United States of America; 2 Department of Pediatrics, University of Cincinnati College of Medicine, Cincinnati, Ohio, United States of America; Central Queensland University, Australia

## Abstract

**Background:**

Obstructive Sleep Apnea (OSA) is associated with medical and neurobehavioral morbidity across the lifespan. Positive airway pressure (PAP) treatment has demonstrated efficacy in treating OSA and has been shown to improve daytime functioning in adults, but treatment adherence can be problematic. There are nearly no published studies examining functional outcomes such as academic functioning in adolescents treated with PAP. This study was conducted as an initial step towards determining whether PAP treatment improves daytime functioning among adolescents with OSA.

**Methods:**

Self-reported academic grades, self- and parent-reported academic quality of life, and objectively-measured attention were assessed before and after PAP was clinically initiated in a sample of 13 obese adolescents with OSA, as well as 15 untreated obese Controls without OSA. Based on adherence data, the treated group was divided into PAP Users (n = 6) and Non-Adherent participants (n = 7).

**Results:**

Though demographically similar, the three groups significantly differed in how their academic performance and attention scores changed from baseline to follow-up. Non-Adherent participants showed worsening functioning over time, while PAP Users showed stable or improved functioning, similar to controls.

**Conclusion:**

Although many adolescents prescribed PAP for OSA are non-adherent to the treatment, those who adhere to treatment can display improved attention and academic functioning.

## Introduction

Obstructive Sleep Apnea (OSA) is a nocturnal breathing disorder associated with neurobehavioral deficits in young children and adults [1], [2]. Less is known about adolescents with OSA, but we recently reported that this group shows attention deficits and diminished academic grades [3]. Young children whose OSA is treated with adenotonsillectomy can show improved daytime functioning, though the long-term impact of pediatric OSA remains unclear [1]. In adults, surgical interventions are less effective, so positive airway pressure (PAP) therapy is often prescribed. PAP therapy requires that patients wear a snug-fitting nasal interface attached to a blower that creates a pneumatic “stent” to maintain airway patency during sleep. When used properly, PAP therapy can dramatically improve OSA in adults [4] and children [5]. However, adherence with PAP therapy is often a concern. This is particularly true when PAP is prescribed for adolescents with OSA, many of whom are obese and therefore unlikely to achieve cure via adenotonsillectomy [6]. Adolescents are notorious for diminished adherence to medical regimens and, indeed, adolescents prescribed PAP for OSA tend to have poor treatment adherence [7].

Adults who adhere to PAP therapy show gains in daytime functioning, including improved alertness, quality of life, and at least some aspects of neurobehavioral functioning [4], [8]. To our knowledge, only one published study has examined the impact of PAP therapy on the daytime functioning in children with OSA; Marcus and colleagues demonstrated improved daytime sleepiness after 6 months of PAP treatment, but no change in other functional outcomes, including behavior, temperament, and school performance [5]. That study did not specifically assess attention, did not differentiate participants who were adherent to treatment from those who were not, and used a school performance measure that was of unproven sensitivity to OSA. These methodological factors might have obscured changes in functioning for those individuals who were more adherent to PAP.

Given the scarce evidence supporting PAP effectiveness in improving daytime functioning in adolescents, clinicians can find it challenging to convince already skeptical adolescents of the potential benefits of PAP therapy, which requires time to establish and diligence to maintain. Instead, surgical options that do not require long-term patient adherence are often undertaken, despite research to suggest that surgery has lower success and higher complication rates in obese adolescents, a rapidly-growing population at very high risk for OSA [6], [9], [10]. If the field is to improve care, large-scale investigation of the impact of PAP therapy on OSA-linked morbidity is needed. However, sample size limitations make it difficult to publish the preliminary studies needed to justify the expense of larger-scale work. Even Marcus et al.’s study, conducted over 3 sites, reported on only 21 subjects [5]. Other studies of pediatric PAP adherence have had larger samples, but most subjects had a heterogeneous mix of neurodevelopmental conditions [7], [11] which effectively ruled out standardized assessments of change in daytime functioning.

The current single-site study was designed to build the case for larger-scale work in this area, and to help inform clinical care. This paper examines whether PAP improves academic functioning and attention among adolescents who have OSA related to obesity, not to a neurological or developmental disorder that could independently impact daytime functioning. We prospectively studied the functioning of obese adolescents with OSA who were clinically prescribed PAP therapy, as well as untreated obese controls without OSA. We hypothesized that adolescent PAP users would show a more positive change in academic performance and attention as compared to those nonadherent to PAP, and to untreated controls.

## Methods

### Ethics Statement

All methods were approved and overseen by the Institutional Review Board at Cincinnati Children's Hospital Medical Center. After receiving print and verbal information regarding the study, all participants provided written documentation of informed assent and parents of participants provided written documentation of informed consent.

### Participants

The 35 recruited participants were a subset from a study of neurobehavioral functioning in overweight 10–16-year-olds with and without OSA [3]. All were obese and were without a history of neurological illness/injury, neurodevelopmental disorder, condition involving daytime hypoxia, or ongoing psychiatric medication use. After a baseline inpatient polysomnogram (see [3]), participants who showed evidence of OSA were referred for clinical follow-up. For the current study, we enrolled 18 patients who were clinically diagnosed with OSA and who, at their doctors’ prescriptions, had PAP therapy titrated during a second overnight polysomnogram to optimally correct obstructive respiratory events, respiratory arousals, and excessive work of breathing. To account for practice and exposure effects, we also recruited a ***Control*** Group of 17 obese participants who did not have OSA and consequently received no airway treatment. Of those enrolled, 5 patients prescribed PAP and 2 controls were lost to follow-up due to subjects moving out of the region (1 control, 1 prescribed PAP) or the family not responding to multiple calls and letters. This left 13 patients prescribed PAP and 15 controls for analyses.

For the patients whose PAP therapy was clinically-prescribed, PAP machines and interfaces were supplied by various medical equipment providers depending on each patient’s location and insurance provider. At the time of this study, the PAP units varied, so there was no consistent objective index of treatment adherence. In order to obtain a consistent measure of PAP adherence across patients, we called parents monthly for 4 months after starting treatment to ask how many nights per week the participant had used PAP, the number of hours of PAP use during nights it was used, and the hours of nightly sleep typically obtained. Information from the month 3 and 4 reports was used to estimate adherence for this study. Adherence rates were calculated as the percent of sleep time during which PAP was used. Assessed in this way, adherence rates were quite variable across participants, ranging from 0–88%. Because we were interested in evaluating functional outcomes in participants who were reported to be more adherent with PAP treatment relative to those who were less adherent, we split the treatment group at the median of 21%, resulting in a ***Non-Adherent*** Group (n = 7) and a ***PAP Users*** Group (n = 6).

### Measures

All participants underwent neurobehavioral evaluations at baseline and at 4-months after PAP initiation, or an equivalent time period for controls. The 4-month follow-up point was set to allow adequate time for stable adherence patterns to develop and to exceed the length of the conventional 10- to 12-week academic grading period in the United States (US) school system. In fact, because at our center at the time of this study there was a delay between initial OSA diagnosis and initiation of PAP treatment, the follow-up evaluation occurred on average 8.6 months after the baseline assessment (range: 6–15 months).

The neurobehavioral evaluation was comprised of office-based tests and parent- and self-report questionnaires. For the purposes of the present study, we focused on measures that (a) were repeated across both assessment time points and (b) addressed constructs found to be vulnerable to OSA in our baseline, cross-sectional study [3]. ***Academic Performance*** was assessed in two ways. First, subjects self-reported their grades based upon the US convention of using the letters “A” through “F”, in which “A” grades denote the highest marks and “F” grades denote failing marks. To allow for variation in marks across subjects and to increase meaningful variance while retaining a single outcome variable, we adopted an 8-option scale based upon Wolfson and Carskadon’s epidemiological work [12] that allows for “mixed” grades (e.g., “mostly A”, “A and B”, “mostly B”, “mostly B and C”, etc) and that was then converted to the US 4-point convention (A = 4.0, A and B = 3.5, B = 3.0, etc; see [3]). Second, we obtained parent- and self-reported scholastic functioning on the well-validated Pediatric Quality of Life measure (Peds-QL 4.0 Generic) [13]. ***Attention*** was assessed via the age-normed Total Correct z-score on the computerized Gordon Diagnostic System, a validated sustained attention test [14]. For this task, test-takers view a screen on which single-digit numbers are flashed at 1 second intervals for 9 minutes; test-takers are asked to push a large button immediately after the second number of a pre-specified 2-number series, and to refrain from responding at other times.

## Results

**Figure 1:**
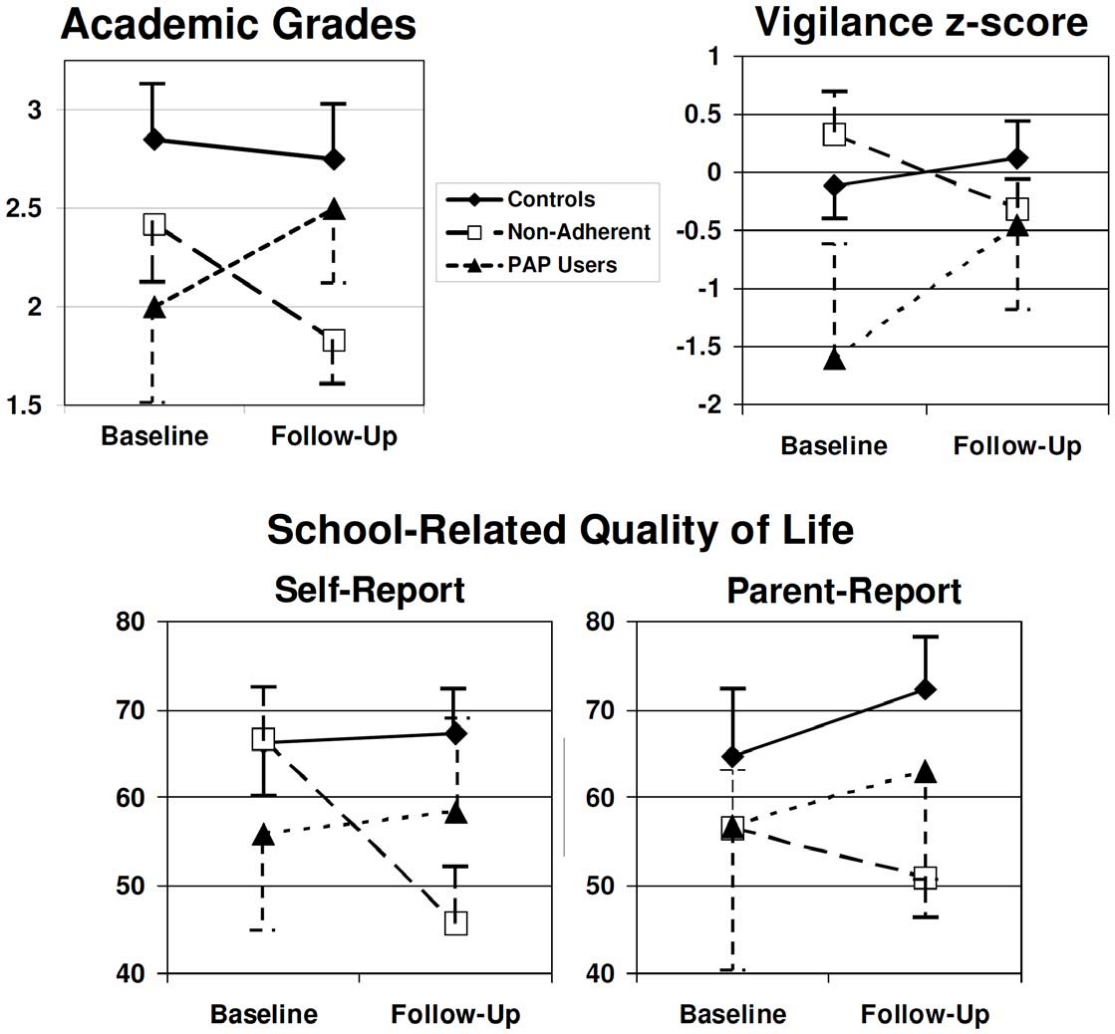
Change over time in sustained attention/vigilance and academic performance among adolescents who used PAP, those who were non-adherent to PAP treatment, and untreated controls. Error bars reflect the standard error of the mean. Higher scores denote better functioning. Self-reported academic grades are expressed according to the US 4-point convention (max  = 4.0; see [3]). Vigilance is expressed as an age-adjusted z-score compared to published norms [14]. Academic quality of life is expressed on the 0–100 scale used by the Peds-QL, on which completely healthy individuals average around 80 [13].

Chi-square and Kruskal-Wallace tests were used to compare all three groups and to contrast the two PAP-prescribed groups. Non-parametric tests were conservatively used because of the small sample and concerns about violated assumptions of parametric statistics. As shown in Table 1, the three groups did not significantly differ on sex, race, age, baseline scholastic functioning or vigilance, or time between baseline and follow-up assessments. The two PAP groups also did not differ in severity of OSA or obesity. As predicted, the PAP Users Group displayed a more favorable change than the Non-Adherent Group across all four outcome measures (Table 1). Although parent questionnaire findings fell just short of the significance threshold of 0.05, they followed the same pattern as the other outcome measures. As illustrated in Figure 1, the Non-Adherent Group showed a decline in school performance and attention from baseline to follow-up, while the PAP Users Group displayed stable or improved scores over time, similar to Controls. All of the PAP Users had stable or improved grades, while about half of the Non-Adherent group’s grades declined. 83% of PAP Users had improved attention over time, whereas 86% of Non-Adherent participants’ attention worsened.

**Table 1:** Cross-Group Comparisons on Descriptive Variables and Change Over Time in School Performance and Attention.

	Group			Difference across all 3 groups?		Non-Adherent vs PAP Users	
	Controls	Non-Adherent	PAP Users	χ^2^ (df = 2)	*p*	χ^2^ (df = 1)	*p*
**Descriptive Variables**							
% Boys	33	71	67	3.62	.164	.03	.853
% Caucasian	20	14	50	2.63	.269	1.94	.164
Age (yrs)	13.3±1.9	14.4±1.5	14.8±1.8	2.78	.250	.18	.668
Baseline Body Mass Index (BMI)	34.9±4.4	45.4±15.1	42.4±6.1	6.13	.047	.00	.999
Baseline BMI z-score for age and sex	2.36±0.18	2.64±0.28	2.64±0.17	8.91	.012	.02	.886
Baseline Obstructive Index	1.7±1.2	9.3±5.7	10.0±6.8	11.48	.003	.02	.886
Baseline Self-Reported Grades	2.8±1.0	2.4±0.9	2.0±1.3	2.47	.291	.32	.572
Baseline Self-Reported School QOL	66.3±22.5	66.7±12.5	55.8±28.7	.63	.728	.32	.572
Baseline Parent-Reported School QOL	64.7±27.5	56.4±16.8	56.7±40.7	.57	.751	.00	.999
Baseline Age-Normed Vigilance (z)	−0.11±1.26	0.32±0.79	−1.61±2.59	3.83	.148	3.07	.080
Weeks Baseline to Follow-up	38.0±6.7	35.7±9.6	36.4±15.6	2.82	.244	.33	.568
Optimized PAP Pressure	---	10.0±2.7	9.2±2.2	---	---	.19	.665
% PAP Adherence	---	5.9±7.8	56.8±22.3	---	---	9.10	.003
**PRIMARY OUTCOMES:** **Change in School Performance and Attention (Positive Values Indicate Improvements from Baseline to Follow-up)**							
Change in Self-Reported Grades	−.10±.60	−.59±.81	.50±.45	7.18	.028	6.25	.012
Change in Self-Reported School QOL	2.3±14.3	−15.8±13.2	2.5±10.8	6.24	.044	4.47	.034
Change in Parent-Reported School QOL	7.7±17.2	−9.2±13.9	12.5±17.1	4.79	.091	3.73	.054
Change in Age-Normed Vigilance	.23±1.4	−.64±.75	1.2±1.5	6.68	.035	3.78	.052

Group data refer to percents for sex and race, and mean ± standard deviation for all others. Body Mass Index (BMI)  =  (mass in kg)/(height in m)^2^. Age- and sex-adjusted BMI conversion made per US Centers for Disease Control and Prevention. Obstructive Index  =  OSA severity as indexed by the number of obstructive apneas + hypopneas per hour of sleep (see [3]). QOL  =  Quality of Life.

## Discussion

The present findings represent an important step in demonstrating the potential efficacy of PAP intervention in reversing two of the most prominent daytime effects of adolescent OSA: inattention and poor school performance. Impressively, a group of adolescents with even modest levels of PAP adherence (57% on average) displayed stable or improved attention and school performance, whereas a similar group of non-adherent adolescents trended towards declines in academic and school performance. This potential utility of even partial adherence to PAP therapy mirrors adult findings, in which improved sleepiness and quality of life have been observed in groups in which PAP use averages around 50% of each night [4], [8]. It remains unknown whether, beyond such group averages, greater individual PAP usage results in greater gains in daytime functioning.

Present findings extend prior work that suggested an improvement in daytime sleepiness after PAP treatment in pediatric populations [5]. There are multiple potential mechanisms by which such gains might occur. When titrated properly and used consistently, PAP markedly reduces or eliminates the sleep disruption and gas exchange abnormalities that characterize OSA [5]; both have been proposed to be key mechanisms that underlie OSA-related neurobehavioral morbidity, and there is evidence to support both as sufficient but not necessary causes of such morbidity [1]. Attempts within the current sample to quantify sleep quantity and quality using objective actigraphy before and after treatment were regrettably unsuccessful because of missing data related to poor adherence with wearing the actigraph units at one or both measurement time points.

Although present results provide important evidence that can be used to support and justify future larger-scale research, findings are considered preliminary. The sample was small, in part due to substantial loss of subjects to follow-up. This has also been observed in the limited other studies in this area [5], suggesting that future work will need to institute a highly comprehensive subject retention protocol or an initial “run-in” period (e.g., demonstration of PAP use at sub-optimal pressure prior to titration) to screen prospective subjects. Our reliance on parent-report of adherence is a further limitation, though if anything the lack of precision associated with such reports would tend to obscure findings, suggesting that current findings may in fact be conservative. Despite concerns that parent-report might overestimate adherence, PAP adherence still tended to be poor in our sample, similar to or slightly worse than that which has been observed among other adolescent samples via objective monitors [5], [7], [11]. Assignment to the groups was not random, and though the groups did not significantly differ on demographic variables or baseline functioning, this may have been due to inadequate statistical power for cross-sectional analyses; in a larger sample, baseline characteristics might prove to be significant predictors of adherence and change in attention or scholastic performance. It is also possible that unmeasured factors (e.g., family disorganization) could confound results, or that adolescents who experienced initial clinical improvements became more motivated to maintain or increase PAP usage. An experimental trial, in which children with known OSA are randomized to a PAP versus placebo (“sham-PAP”) condition could help to eliminate confounds, but would still need to deal with issues of adherence in the treatment arm and would further require careful ethics consideration.

Although these limitations suggest a need for study replication and extension in larger-scale studies, present findings may have important clinical and public health implications. Adolescent obesity has reached epidemic prevalence, placing many adolescents at high risk for OSA [10] while simultaneously reducing the success and increasing the complication rates associated with airway surgery [6]. The scholastic impact of untreated OSA may be of particular concern during adolescence, when school performance more clearly predicts long-term outcomes than at any prior developmental stage [3]. Conversely, successful treatments during adolescence may have particularly high long-term yield [15]. Findings from this study are consistent with clinical recommendations for PAP treatment in adolescents with OSA [5], [11], and add an empirical foundation for clinicians to use while communicating the importance of PAP therapy adherence to patients and their families.

Because PAP adherence rates are low and adolescents who are non-adherent remain at risk for significant medical and neurobehavioral morbidity, it is imperative that future research identify the key obstacles to PAP adherence among adolescents. Considerable progress has been made in identifying predictors of PAP adherence in adults [16]. However, such predictors are likely to be unique in pediatric populations due to developmental factors, including the adolescent transition from parent-directed care to patient-directed care. Based upon the minimal pediatric data available, neither demographic factors nor the mode of PAP delivery (single-level versus bi-level) appear to be major adherence predictors, though some aspects of pre-treatment disease severity may predict adherence [5], [11]. In adolescents, correlates of PAP use remain largely unexplored [16]. Present results, which suggest that adolescent adherence levels are related to changes in functional outcomes, highlight the need for additional work in this area so that novel therapies that promote PAP adherence can be developed and tested.
